# Evaluation of the Effectiveness and Safety of the Use of CODUBIX^®^ ŻEBRA, CODUBIX^®^ S ŻEBRA Rib Bone Prostheses

**DOI:** 10.3390/jcm15135297

**Published:** 2026-07-07

**Authors:** Tadeusz Orłowski, Marcin Zieliński, Janusz Włodarczyk, Piotr Kasprzak, Magdalena Tokarska, Kaja Jezierska, Witold Sujka

**Affiliations:** 1 Institute of Tuberculosis and Lung Diseases, 01-138 Warsaw, Poland; 2 The Respiratory Hospital in Zakopane, 34-500 Zakopane, Poland; 3 Department of Thoracic and Surgical Oncology, John Paul II Hospital, 31-202 Cracow, Poland; 4 Department of Thoracic Surgery, Jagiellonian University Collegium Medicum, 31-008 Cracow, Poland; 5 Department of Neurosurgery, Medical University of Lodz, 90-549 Lodz, Poland; 6 Faculty of Textiles and Design, Institute of Architecture of Textiles, Lodz University of Technology, 116 Zeromskiego St., 90-543 Lodz, Poland; 7 Tricomed S.A., Świętojańska 5/9, 93-493 Lodz, Poland

**Keywords:** thoracic surgery, chest wall reconstruction, Codubix^®^ RIBS, rigid method, hammock method

## Abstract

**Background:** The Codubix^®^ ŻEBRA and Codubix^®^ S ŻEBRA prostheses (by Tricomed SA), made of a biocompatible polypropylene–polyester braid, were developed as tools for the treatment of bone defects resulting from cancer surgery or mechanical injuries. **Methods:** The retrospective analysis investigation presents the efficacy and safety of rib bone prostheses made of knitted polyester–polypropylene fabric used to fill defects in the ribs and sternum. Data were collected from 113 patients (68 males, 45 females) undergoing surgery at three clinical centres. Prosthesis implantation was performed to bridge bone defects in the ribs and/or sternum. The analysis included preoperative and intraoperative data, two follow-up visits and a final interview with the patients. All prostheses were implanted using two techniques for filling defects in the chest wall: the ‘hammock’ (suspension) fixation method in 87 patients, and the ‘rigid’ fixation method in 26 patients. **Results:** The main cause of defects was cancer surgery (95.6%) performed in cases of sarcomas, squamous cell carcinomas, and desmoid tumours. Other causes (e.g., congenital defects and mechanical trauma) were less common. The ‘rigid’ fixation method extended the surgery time by approximately 16 min compared to the suspension method. Differences were also noted in the recovery period—an average of 56 days for the ‘hammock’ method and 30 days for the ‘rigid’ method. During the second follow-up visit, the treatment outcome using these prostheses was rated as good in 90.3% of cases. The average duration of hospitalisation was 21 days, regardless of the implantation method. No prosthesis-related adverse events were reported. Complications were observed in 21 cases in the first days after surgery. The most common ones were sensory disturbances (5.3%), infections (3.5%), haematomas and blood effusions (2.7%). **Conclusions:** A retrospective study demonstrates that knitted prostheses are safe and effective solution for repairing extensive defects resulting from tumours, trauma or congenital malformations. The implants ensure high patient comfort and maintain normal physical functioning without interference.

## 1. Introduction

Resection of a portion of the chest wall followed by reconstruction is used in the treatment of chest wall diseases caused by various causes, such as tumours, congenital defects, or trauma. Other causes include infectious inflammatory processes and radiation-induced wounds. In these cases, the extent of resection is the most important factor influencing the functional outcome of surgery in cases of primary and metastatic tumours [1,2,3].

Chest wall reconstruction was first described by Tensini in 1906, when a pedicled latissimus dorsi muscle flap was used to close a defect in the anterior part of the chest [4]. Reconstructive techniques have progressed significantly since then thanks to advances in surgery and the introduction of a wide range of prosthetic materials and bioprostheses. Chest wall defects typically result from the resection of primary tumours, metastatic lesions, or locally invasive tumours [5]. The method of reconstruction of the chest wall frame depends on the defect location, the number of missing ribs, and the size of the defect [6]. Chest wall reconstruction should restore its stability, protect internal organs, maintain normal respiratory function, and ensure an appropriate appearance without restricting normal growth in younger patients.

A prosthetic material for chest wall reconstruction should meet a number of criteria. It should be malleable enough to be easily moulded and conformed to the required shape, but it is also important that it maintains sufficient stiffness to eliminate paradoxical movement of the chest. The material must also be physically and chemically inert and capable of integrating with the patient’s tissues [7]. Furthermore, it should be highly resistant to infection, insensitive to X-ray radiation, and affordable. Commonly used non-biological materials for chest wall reconstruction include metals (primarily titanium), synthetic materials (polypropylene, nylon, silicone, polytetrafluoroethylene), and ceramic implants. Auto- and alloimplantation methods (e.g., rib transplantation) and the use of stem cells are also possible [8,9].

Reconstruction of oncological chest wall defects is a significant challenge and typically requires the collaboration of surgeons from various specialties to restore both the patient’s appearance and function. Advances in thoracic oncology have expanded the range of indications for surgical procedures in patients with locally advanced cancers [10]. Not all chest wall defects require reconstruction. Typically, small defects (<5 cm) or resections involving fewer than three ribs do not require reconstructive procedures, and the integumentary tissues are sufficient to support the chest wall defect. Posterior chest wall defects up to 10 cm in size may also not require reconstruction because the scapula and supporting muscles provide sufficient support to the affected area [11,12]. In the case of lung cancer infiltration of the chest wall, it is necessary to remove a section of one normal rib above and below the infiltration border. The anterior and posterior resection margin should be at least 3–4 cm in the case of a primary chest wall tumour. A defect of 2–3 ribs, especially in the posterior or upper thoracic region, usually does not require special treatment and the defect is covered by soft tissue and the scapula. For larger defects, it is necessary to restore chest wall stability to prevent paradoxical movements and possible subsequent respiratory failure. Biological materials most commonly used for reconstruction include pedicled muscle flaps and myocutaneous flaps, omentum flaps, bone grafts (from the ribs, fibula, iliac crest), autologous or preserved grafts of the wide thigh fascia, pericardium and dura [13]. The aim of the analysis was to assess the effectiveness and safety of use of knitted polypropylene–polyester implants for filling defects in the chest wall (Codubix^®^ ŻEBRA, Codubix^®^ S ŻEBRA, Tricomed SA, Lodz, Poland) and to compare two methods fixating the prostheses—‘hammock’ and ‘rigid’ fixation.

## 2. Materials and Methods

### 2.1. Description of the Study

A study of patients submitted for surgical repair of chest wall defects was conducted retrospectively. Data collected from three research centres (Institute of Tuberculosis and Lung Diseases in Warsaw, Specialist Hospital for Lung Diseases in Zakopane, and John Paul II Krakow Specialist Hospital in Krakow) were analysed in detail. Information was obtained regarding the surgical process and the patients’ well-being after the procedures (two follow-up visits: up to 6 months and after 6 months, and a patient interview).

The retrospective analysis enrolled 113 patients (68 men, 45 women) from three research centres. Each patient had complete medical records collected during the initial diagnostic examination and two follow-up visits—up to 6 months after the procedure and 6 months after. The hospital database provided demographic information (age, sex, medications, medical history), preoperative information (diagnosis), intraoperative information (type of anaesthesia, surgical technique, prosthesis used, defect size, duration of surgery, complications, adverse events, need for drainage), and postoperative information (hospitalisation time, recovery period, complications within the first 24 h after surgery, implant-related adverse events). The surgeries took place between 1997 and 2024. Each patient received a non-sterile ribs prosthesis.

During two follow-up visits and patient interviews, information was obtained regarding postoperative complications, implant-related adverse events, patient comfort ratings, functional self-assessments, pain level on the VAS scale and foreign body sensation. The surgeries were performed electively, under general anaesthesia, and surgical drain was used.

Follow-up visits were also allowed in order to assess the safety and effectiveness of the implant.

### 2.2. Tested Population

**Figure 1:**
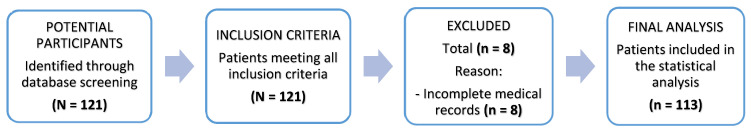
Patient selection flow diagram.

Initially, a total of 121 patients who underwent surgical treatment during the study period were identified. We excluded 8 patients due to the following condition—incomplete medical record. The final study cohort consisted of 113 patients, aged 18 to 83. Patient selection process is illustrated in the STROBE diagram (Figure 1). This group represents the total population of undergoing individuals from three research centres who were qualified for the procedure and possessed complete clinical documentation collected during and after the surgery, as well as after two follow-up visits, which occurred within 6 months and at least 6 months after surgery. Due to the requirement of comprehensive records for long-term evaluation, only patients with a full dataset were analysed. Consequently, no significant missing data were encountered for the primary variables analysed in this study. They were submitted for thoracic bone resection with chest wall reconstruction using a rib bone prosthesis. We determined the potential exclusion criteria as follows: incomplete medical records, liver cirrhosis, thrombocytopenia (platelet count < 100 × 109/L), chronic renal failure, and the use of immunosuppressive and steroid medications. Pregnant patients were also subject to exclusion from surgeries where applicable.

### 2.3. Product Description

The chest wall defect prostheses are made using a knitting technique and two multifilament yarns: polypropylene 94% and polyester 6%. It has a low specific weight and a low melting point, which gives the prosthesis the necessary hardness and stiffness. Polyester yarn ensures its resistance to bending and pressure, and protects it against spreading of the surface. After the knitting process, a left-right weave braid in the form of a sleeve is created, which is then used for the further production process.

The products are available in two variants—non-sterile and sterile. Rib implants are manufactured in four sizes (RZ-1–RZ-4). In addition, sternum prostheses are available in eight sizes (RZ-5–RZ-12). The implants are made provide them with high resistance, low weight and hydrophobicity and contribute to the non-absorption of liquids and physiological fluids. These features, as well as the absence of chemical activity, non-toxicity and a high degree of healing, mean that the prostheses are similar in terms of physical properties to the patients’ natural bones.

**Figure 2:**
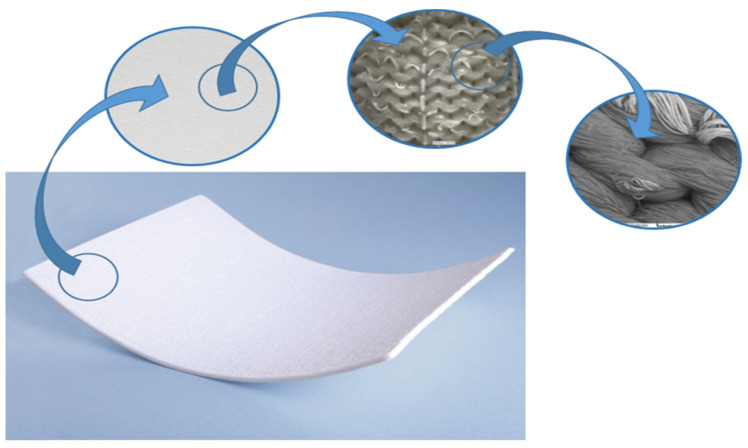
Photo of the braid taken with a scanning electron microscope (SEM).

**Figure 3:**
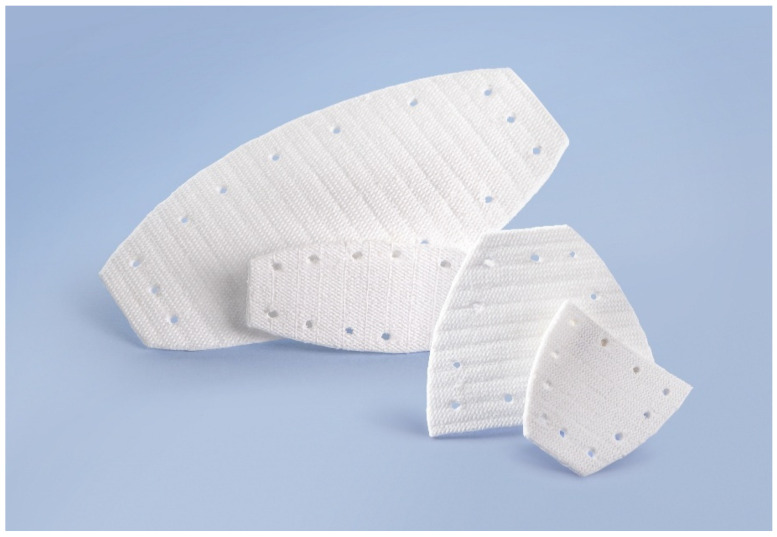
Prostheses used to treat defects of the sternum.

Figure 2 shows images of the braid taken with a scanning electron microscope (SEM), while Figure 3 shows images of prostheses used to treat defects of the sternum. Tables S1 and S2 show the physical and chemical characteristics of the implanted prostheses are attached in Supplementary Material.

### 2.4. Surgical Techniques

Implanting of the prostheses was carried out using two methods: the traditional ‘rigid’ method and the ‘hammock’ suspension method.

**Figure 4:**
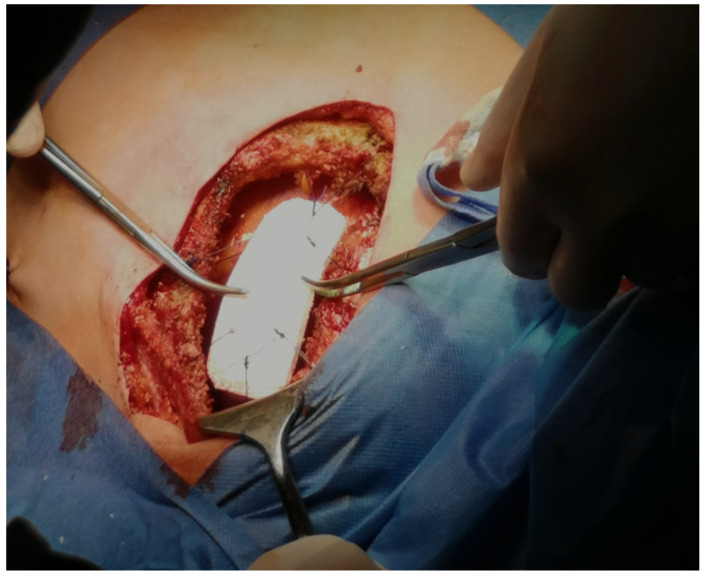
Intraoperative photo—implantation of Codubix^®^ RIBS prosthesis.

In the rigid technique group the size of the defect was similar to the size of the prosthesis. In the hammock method the selected prosthesis was smaller than the defect to be treated. The explanation of this policy is that the defect shrinks during the late postoperative course, which, for a prosthesis with a surface area equal to the defect, could exacerbate pain and cause the prosthesis to protrude, which might cause painful ulceration of the chest wall. This complication does not occur if the prosthesis is smaller than the defect of the chest wall after resection. Covering of the prosthesis with a muscle flap or the omental flap additionally protects the reconstructed chest wall. Figure 4 illustrate the surgical steps for the implantation of the prosthesis using the hammock fixation method. Table 1 shows the comparison between different surgical methods.

**Figure 5:**
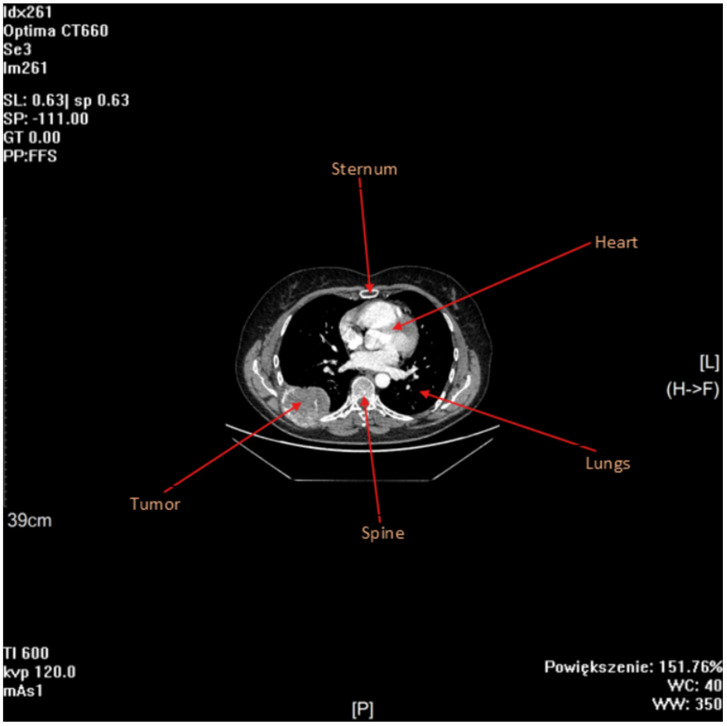
Chest CT scan before tumor resection.

**Figure 6:**
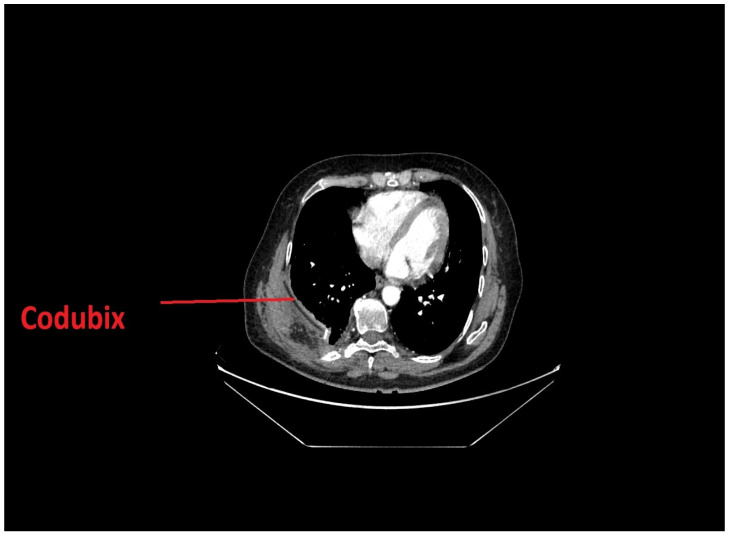
Chest wall CT scan after prosthesis implantation.

Figure 5 and Figure 6 shows CT scan of preoperative and postoperative chest CT in female patient age 55 with diagnosis of chondrosarcoma of the chest wall. Operative procedure—chest wall resection including ribs V-VIII on the right side with wedge resection of the right lower pulmonary lobe. The arrow indicates the prosthesis and anatomical structures.

### 2.5. Statistical Analysis

The following tests were used to analyse the data for statistical significance: Mann–Whitney U test, Pearson’s Chi-square test and the Chi-square test with Yates Correction (for bivariate tables). The significance level was set at α = 0.05. A *p*-value was calculated from the test results and compared with the established α value. The null hypothesis was rejected when the *p*-value was lower than 0.05.

The non-parametric Mann–Whitney U test was used to analyse the numerical data to compare two independent groups. For this test, the measure of central tendency is the median. Non-parametric tests are used instead of one-way analysis of variance when the data do not meet the assumptions of a normal distribution of the dependent variable and homogeneity of variance between groups.

For qualitative data, bivariate tables and non-parametric tests such as Pearson’s chi-square test (for n × m tables) and the chi-square test with Yates correction (for 2 × 2 tables) were used to test whether there was a statistically significant relationship between two qualitative variables.

## 3. Results

### 3.1. Analysis of Preoperative and Immediate Postoperative Information

A non-parametric Mann–Whitney U test was used to assess the effect of the surgical method—hammock and rigid—on the duration of surgery, hospitalisation time and recovery period. A significance level of 0.05 was adopted. The data are shown in Tables S3–S5. All the tables are attached in Supplementary Material.

The duration of surgery was comparable between the hammock and rigid techniques. The average duration of hammock surgery was 217 min, median: 225 min, and of the rigid surgery 223 min, median: 240 min.

Marked variations in hospitalisation times were noted when comparing the hammock technique to the rigid method. Average hospitalisation time for the hammock method: 23 days, median: 18 days; average hospitalisation time for the rigid method: 14 days, median: 11 days.

A significant difference in hospitalisation length was noted when comparing the hammock method to the rigid method (*p* < 0.05). The average recovery period for the rigid method was 8 weeks, median: 8 weeks, and for the hammock method 4 weeks, median: 4 weeks.

Early post-operative complication rates were comparable between the hammock and rigid techniques. Table S6 shows the incidence of complications on the first postoperative day. Table is attached in Supplementary Material.

### 3.2. Analysis of Information Collected During the First (Up to 6 Months) and Second (After 6 Months) Visit

Bivariate tables and Pearson’s chi-square non-parametric test were used to assess the impact of the surgical method—hammock or rigid—on complications, adverse events, efficacy/safety ratings, comfort/discomfort ratings, foreign body sensation up to 6M and after 6M; a significance level of 0.05 was adopted. A statistical analysis of the data is presented in Tables S7–S10 and Figures S1–S3. All the tables and figures are attached in Supplementary Material. There were no significant differences in complications up to 6 months and at the 6-month follow-up between the hammock and rigid method.

No complications occurred in 77.01% of cases in the hammock method group, and in 61.54% in the rigid method group. Complications occurred in 14.94% of cases in the hammock method group, and in 34.62% in the rigid method group. Adverse event rates within the first 6 months were similar between the hammock and rigid techniques, showing no significant variation.

At the 6-month follow-up, the incidence of adverse events differed significantly between the hammock and rigid methods (*p* < 0.05). In terms of safety, 88.51% of cases in the hammock group experienced no adverse events, compared to 69.23% in the rigid group.

### 3.3. Confidence Intervals of the Mean Values

The 95% confidence intervals for the mean values were determined considering all patients (hammock+rigid) and divided into hammock-operated and rigid-operated groups. Information is also provided on the number of cases analysed (N-valid) and the median and standard deviation. The confidence intervals are shown in Tables S11–S13. All the tables are attached in Supplementary Material.

### 3.4. Demographics

The study involved patients from three research centres: 26 patients from Centre I (15 women, 11 men), 70 from Centre II (25 women, 45 men) and 17 from Centre III (5 women, 12 men). The demographics, as well as the patients’ pre-operative status (including diagnosis), are shown in Table 2.

### 3.5. Perioperative Information

All surgeries were carried out under general anaesthesia with usage of surgical drain. The average duration of the surgery was 220 min. During the procedures, there were no adverse events related to the implant used; however, intraoperative complications, including the fistula, bleeding, wound leakage or respiratory failure, were observed in seven out of 113 patients. During the procedures, patients received only one prosthesis variant: non-sterile prostheses. Table 3 provides intraoperative information.

### 3.6. Post-Operative Information

The average length of hospitalisation of patients was about 21 days. During the immediate postoperative phase, several complications were recorded, ranging from systemic and biochemical disturbances (such as iron deficiency and impaired coagulation) to localised surgical site issues, including haematomas, effusions, and infections. Notably, two deaths occurred during the follow-up period due to cardiovascular and respiratory complications (bronchiopleural fistula); however, neither event was found to be related to the implanted prostheses. Patient-reported outcomes shortly after surgery indicated high tolerability, with low average pain scores on the VAS scale and minimal reports of general discomfort. More detailed information is included in Table 3.

### 3.7. Post-Operative Evaluation

Two follow-up visits were scheduled—up to 6 months and after 6 months after the procedure—during which patient comfort, and the safety and effectiveness of the implants were assessed.

The first follow-up visit took place 90 days after the surgery on average. While postoperative complications were observed in a subset of the cohort, serious implant-related adverse events and subsequent prosthesis removals remained rare (Table 4). The efficacy and safety of the implants were assessed as good in the majority of patients. Regarding patient comfort, although minor sensory issues such as foreign body sensation and localised discomfort were reported, the average pain levels remained low on the VAS scale (Table 4).

The second visit took place on average 319 days after the procedure. Late postoperative complications were observed in a small proportion of the cohort, including a few cases of oncological recurrence. While further prosthesis removals were necessitated at this stage—primarily due to localised infections, abscesses, or inflammatory responses—the overall efficacy and safety profile of the implants remained high for the vast majority of patients (Table 4). Regarding patient-reported outcomes, both the average pain levels on the VAS scale and the prevalence of foreign body sensations remained stable and comparable to the results from the first visit, indicating favourable long-term tolerability of the implant (Table 4). Complete data from the first and the second follow-up visit (including postoperative complications, assessment of efficacy and safety, adverse events, number of days post-surgery, assessment of pain sensation, assessment of function/dysfunction, and foreign body sensation) are described in Table 4.

The last, optional follow-up visit—interviewing patients longer after reconstructive surgery—was conducted after 1568 days or approximately 4.5 years on average Among the returning cohort, clinical outcomes remained generally positive and stable over this extended period. While a few instances of late-stage prosthesis removal and minor discomfort were documented, the majority of patients reported no implant-related complaints and maintained consistently low pain levels on the VAS scale. Details of the patient interview are included in Table 5 and comfort ratings are pictured in Table 6.

## 4. Discussion

Removal of part of the chest wall is a common surgical procedure used to treat a variety of diseases—malignant tumours, congenital malformations and chest trauma. In such cases, it may be necessary to remove one or more ribs, which significantly affects the anatomical cohesiveness of the thorax and its proper functioning. The increasing incidence of rib fractures is associated with the ageing of the population and increased vulnerability of bones to injuries resulting from degenerative conditions (e.g., osteoporosis). Bone-replacement techniques for rib reconstruction are being developed, with the main aim of preserving the patients’ respiratory function [14]. Prostheses made from a variety of materials, including metals such as titanium (mesh, plates) or polymers (e.g., polyetheretherketone PEEK, polypropylene PP, polyester PE), are used to reconstruct the chest wall. A study conducted by Pikin et al. [15] on a group of 22 patients at four research centres confirmed that reconstruction with the prosthesis—manufactured from polypropylene/polyester braid [16]—resulted in a satisfactory post-transplant outcome and aesthetic result. This material is easy to use and is associated with a low number of complications. A study conducted by Appel et al. [17] on a cohort of 11 patients confirmed the high clinical efficacy of prostheses in chest wall reconstruction. The authors achieved excellent functional outcomes, characterised by low pain intensity (mean NRS 1.7) and complete chest wall stability with no paradoxical motion observed on dynamic MRI. A low incidence of complications directly related to the implant (9.1%) suggests that this material represents a safe and user-friendly alternative to traditional surgical techniques. Technological advances have led to an increase in the popularity of creating customised implants using 3D printing. An example of the use of 3D-printed titanium prostheses was described by Goldsmith et al. [18]. The described patient underwent extensive resection of two ribs and half of the sternum due to a malignant tumour. A titanium prosthesis made using 3D printing technology was developed based on CT scans. Another example is the use of polyetheretherketone (PEEK) implants made using 3D printing for skeletal reconstructions in the chest wall. A study conducted by Wang et al. [19] on a group of 18 patients showed that these implants are both effective and safe to use. Some limitation was encountered due to the use of solely 3D implants manufactured only from PEEK. However, the production time for polyetheretherketone implants was found to be shorter than for prostheses manufactured from titanium. Another limitation is that 3D PEEK implants have similar tensile and flexural strengths comparable to the sternum and ribs. They have low elasticity, which results in minimal disruption of the thorax’ mobility. Also, the follow-up period was so short (6–12 months) that the results may not be considered fully reliable.

Currently, no published studies have directly evaluated the hammock technique against the rigid method for rib prosthesis fixation. Consequently, this study serves as one of the few available reference points for comparing these two surgical techniques.

Our findings highlight distinct clinical profiles for both rib prosthesis fixation techniques evaluated across the participating centres. The rigid method was associated with slightly extended operative times but offered a markedly accelerated convalescence period compared to the hammock technique. Nevertheless, both approaches demonstrated therapeutic equivalence regarding patient quality of life, with the overwhelming majority of individuals achieving optimal comfort, minimal residual pain, and successful functional restoration.

Several limitations of this study warrant consideration. The retrospective design precluded randomisation, potentially introducing selection bias and confounding by indication. Moreover, the extended study period subjects the results of evolving perioperative care standards. Finally, the loss to follow-up in certain cases may limit the robustness of our long-term conclusions. Furthermore, the absence of objective pulmonary function data in this study limits our ability to definitively compare respiratory outcomes and physiological recovery between the two surgical techniques.

A crucial consideration in chest wall reconstruction, particularly when utilising the ‘hammock method’ for large defects, is the maintenance of structural rigidity. Potential concerns regarding this technique include the risk of paradoxical chest wall movement, which can adversely affect respiratory mechanics and lead to prolonged ventilator dependence. Theoretically, it is possible that paradoxical movement following the hammock method can lead to ventilator dependency but there was single case of such complication in the presented group. In fact, there are some paradoxical movements of the chest reconstructed with the hammock method of prostheses implantation but such movements last for a relatively short time not exceeding 2–3 weeks and then subside due to ingrowth of the surrounding tissue into the prosthesis. Spirometric tests were not performed postoperatively and we relied on the clinical course of patients recovery after surgery. There were no respiratory insufficiency patients with exception of two patients who died postoperatively after right pneumonectomy. Any analysis of 2/87 cases of the hammock group would not be meaningful.

Prerequisites for qualifying patients were two follow-up visits and an optional remote interview. Follow-up visits were carried out at intervals of up to six months and a minimum of six months after the operation. They assessed the safety and effectiveness of the prosthesis, as well as the quality of the patients’ wellbeing.

During the long-term follow-up period, postoperative complications were observed in a subset of the cohort, with a minority of patients requiring prosthesis explantation due to wound infection and abscess formation. Late postoperative complications for this type of surgery are quite common. Infections are caused by poor implant protection. An improperly fitted implant is easily damaged, which can lead to its fracture. A lot also depends on the material used to manufacture the prosthesis. Corrosion of the material, allergic reactions or changes in mechanical properties can contribute to pain and even bone or tissue damage. After tumour removal or as a result of an injury, the structure of the chest is changed. In the long term, this causes deformity and reduced mobility in the thoracic region, and impairs normal respiratory function. The implant is a foreign body, which can lead to chronic inflammation. This results in impaired implant stability, pain, discomfort or reduced tissue regeneration capacity [20]. According to the collected data, the hammock technique is characterised by high efficacy and safety, as well as significant postoperative comfort (all rated at 84% during the second follow-up visit).

## 5. Conclusions

The ribs implants are used for the reconstruction of extensive defects of the chest wall. They are manufactured using a knitting technique from polypropylene fibres with the addition of polyester yarn. The material properties allow the implants to adapt to the defect while maintaining mechanical resistance and biocompatibility [21,22]. A retrospective analysis on 113 patients suggests that restoration of extensive defects with the prostheses is a safe and effective solution for defects caused by cancer, congenital malformations or mechanical injuries. The implants did not interfere with the patients functioning. The majority of patients experienced no pain associated with the implantation of the prosthesis. Foreign body sensation was reported by 21 of 113 patients. The average duration of prosthesis implantation surgery for all patients was 220 min, with the rigid method lasting 16 min longer than the hammock method. The collected data indicates that the ‘hammock’ method is more advantageous compared to the ‘rigid’ method. However, the choice of the method depends mainly on the implant site, e.g., in the case of the sternum, can be more convenient to implant the prosthesis using the ‘rigid’ method. In order to obtain more reliable results, further prospective trials with a longer follow-up period should be conducted. Future prospective studies should incorporate longitudinal spirometric assessments as a standardised outcome measure to better quantify the respiratory impact of each approach.

## Figures and Tables

**Table 1:** Comparison of prosthesis fixation techniques in chest wall reconstruction.

Fixation Technique	Biomechanical Characteristics	Main Clinical Advantages	Limitations and Complication Risks
Rigid fixation	Direct, rigid osteosynthesis using hardware (screws, clips, titanium plates).	Maximum initial stability, optimal organ protection, effective elimination of paradoxical chest wall motion.	Restriction of physiological chest wall compliance; high risk of hardware failure, screw pull-out, or implant fracture due to continuous respiratory stress.
Suspension/Hammock technique	Implant suspended on surrounding anatomical structures using heavy non-absorbable sutures, without rigid bone-penetrating elements.	Preservation of natural respiratory mechanics; significant reduction of shear stress at the implant-bone interface; elimination of screw migration risk.	Overall stability relies on suture strength (risk of rupture leading to asymmetry or displacement); lower initial resistance to severe blunt force trauma.
Hybrid/Semi-rigid fixation	Utilisation of varied support points: rigid fixation in stable areas (e.g., sternum) combined with flexible suturing in mobile areas (e.g., lateral ribs).	Optimal biomechanical compromise, effectively balancing central structural stability with the peripheral elasticity required for normal respiration.	Increased technical complexity of the surgical procedure; requires precise material selection and potentially longer operative times.
Sandwich technique (PMMA composite)	A rigid, hand-moulded composite plate created intraoperatively by sandwiching polymethyl methacrylate (PMMA) bone cement between two layers of synthetic mesh.	Highly cost-effective and widely available; provides immediate structural support and excellent organ protection; can be manually adapted to large, irregular defects.	Completely non-compliant, impairing natural respiratory mechanics; impermeable nature increases the risk of seroma and infection; high risk of material fatigue and cement fracture over time.

**Table 2:** Demographic data.

	Codubix^®^ ŻEBRA Prosthesis Implanted Using the ‘Hammock’ Method (*n* = 87)	Codubix^®^ ŻEBRA Prosthesis Implanted Using the ‘Rigid’ Method (*n* = 26)	All Patients (*n* = 113)	*p*-Value
Age (range)	52 (18–83)	53 (20–75)	53 (18–83)	<0.001
Sex				
female	30 (34.5%)	15 (57.7%)	45 (39.8%)	
male	57 (65.5%)	11 (42.3%)	68 (60.2%)	<0.001
Allergy to drugs	-	-	-	
Medical history—Other data				
Taking medication	32 (36.8%)	3 (11.5%)	35 (31.0%)	
Cardiovascular diseases	8 (9.2%)	3 (11.5%)	11 (9.7%)	
Eye diseases	1 (1.1%)	0	1 (0.9%)	
Metabolic diseases	1 (1.1%)	0	1 (0.9%)	
Skin necrosis	1 (1.1%)	0	1 (0.9%)	
Cyst	1 (1.1%)	0	1 (0.9%)	
History of mastectomy	2 (2.3%)	2 (7.7%)	4 (3.5%)	
Enlarged liver	0	1 (3.8%)	1 (0.9%)	
None	45 (51.7%)	13 (50.0%)	58 (51.3%)	
		Diagnosis		<0.001
Injury	0	2 (7.7%)	2 (1.8%)	
Cancer	84 (96.6%)	24 (92.3%)	108 (95.6%)	
Other (congenital rib defect, condition after sternal necrosis)	3 (3.4%)	0	3 (2.7%)	
Pain before surgery				
0	12 (13.8%)	0	12 (10.6%)	
1	8 (9.2%)	22 (84.6%)	30 (26.5%)	<0.001
2	30 (34.5%)	1 (3.8%)	31 (27.4%)	
3	4 (4.6%)	1 (3.8%)	5 (4.4%)	
4	19 (21.8%)	2 (7.7%)	21 (18.6%)	
5	3 (3.4%)	0	3 (2.7%)	
6	8 (9.2%)	0	8 (7.1%)	
8	3 (3.4%)	0	3 (2.7%)	

**Table 3:** Surgery data and post-operative data.

	Codubix^®^ ŻEBRA Prosthesis Implanted Using the ‘Hammock’ Method (*n* = 87)	Codubix^®^ ŻEBRA Prosthesis Implanted Using the ‘Rigid’ Method (*n* = 26)	All Patients (*n* = 113)	*p*-Value
Duration of surgery [min]	217	233	220	0.265
Duration of hospitalisation [days]	23 (4–99)	14 (1–50)	21 (1–99)	<0.001
Antibiotic therapy				
yes	13 (14.9%)	15 (57.7%)	28 (24.8%)	
no	74 (85.1%)	11 (42.3%)	85 (75.2%)	<0.001
Recovery period [weeks]	8 (1–12)	4 (4–8)	7 (1–12)	0.003
Complications during surgery				
Fistula	1 (1.1%)	0	1 (0.9%)	
Bleeding/ haematoma	2 (2.3%)	0	2 (1.8%)	
Wound dehiscence	1 (1.1%)	0	1 (0.9%)	<0.001
Respiratory failure	2 (2.3%)	0	2 (1.8%)	
Stroke	1 (1.1%)	0	1 (0.9%)	
None	80 (92.0%)	26 (100%)	106 (93.8%)	
Complications in the early postoperative period				
Haematoma (local)	2 (2.3%)	0	2 (1.8%)	
Blood coagulation disorders	0	1 (3.8%)	1 (0.9%)	0.180
Local pain	0	1 (3.8%)	1 (0.9%)	
Blood exudate	3 (3.4%)	0	3 (2.7%)	
Local inflammation	4 (4.6%)	0	4 (3.5%)	
Respiratory failure	1 (1.1%)	0	1 (0.9%)	
Stroke	1 (1.1%)	0	1 (0.9%)	
Disorders of superficial sensation	6 (6.9%)	0	6 (5.3%)	
Death	2 (2.3%)	0	2 (1.8%)	
No complications	68 (78.2%)	24 (92.3%)	92 (79.6%)	
Adverse events	-	-	-	
Assessment of pain sensation				
1	23 (26.4%)	2 (7.7%)	25 (22.1%)	
2	41 (47.1%)	10 (38.5%)	51 (45.1%)	
3	1 (1.1%)	10 (38.5%)	11 (9.7%)	
4	15 (17.2%)	5 (19.2%)	20 (17.7%)	<0.001
5	3 (3.4%)	0	3 (2.7%)	
6	1 (1.1%)	0	1 (0.9%)	
7	2 (2.3%)	0	2 (1.8%)	
Assessment of function/dysfunction				
Correct	84 (96.6%)	26 (100%)	110 (97.3%)	<0.001
Incorrect	3 (3.4%)	0	3 (2.7%)	

**Table 4:** Data from the first and the second follow-up visit.

First Follow-Up Visit				Second Follow-Up Visit			
	Codubix^®^ ŻEBRA Prosthesis Implanted Using the ‘Hammock’ Method (*n* = 87)	Codubix^®^ ŻEBRA Prosthesis Implanted Using the ‘Rigid’ Method (*n* = 26)	All Patients (*n* = 113)	Codubix^®^ ŻEBRA Prosthesis Implanted Using the ‘Hammock’ Method (*n* = 87)	Codubix^®^ ŻEBRA Prosthesis Implanted Using the ‘Rigid’ Method (*n* = 26)	All Patients (*n* = 113)	*p*-Value
Number of days post-surgery							
	95 (7–180)	53 (13–138)	90 (7–180)	207 (182–651)	721 (250–3995)	319 (182–3995)	<0.001
Postoperative complications							
Inflammatory reaction around the wound	0	1 (3.8%)	1 (0.9%)	not applicable not applicable			0.397
Local pain	6 (6.9%)	2 (7.7%)	8 (7.1%)				
Blood and serous fluid effusion	0	3 (11.5%)	3 (2.7%)				
Necrotic lesions	0	1 (3.8%)	1 (0.9%)				
Infiltration of the fluid reservoir	0	1 (3.8%)	1 (0.9%)				
Wound dehiscence	0	1 (3.8%)	1 (0.9%)				
Pus accumulation	0	1 (3.8%)	1 (0.9%)				
Prosthesis protrusion	1 (1.1%)	0	1 (0.9%)				
Prosthesis collapse	1 (1.1%)	0	1 (0.9%)				
Subendocardial infarction	1 (1.1%)	0	1 (0.9%)				
Haematoma	2 (2.3%)	0	2 (1.8%)				
Infection	1 (1.1%)	0	1 (0.9%)				
Wound healing disorders	1 (1.1%)	0	1 (0.9%)				
Removal of the implant	2 (2.3%)	1 (3.8%)	3 (2.7%)				
Deformity of the thorax wall	1 (1.1%)	0	1 (0.9%)				
Discomfort and restricted movement	1 (1.1%)	0	1 (0.9%)				0.018
Hernia in the scar	1 (1.1%)	0	1 (0.9%)				
Death	1 (1.1%)	0	1 (0.9%)				
None	60 (67.8%)	16 (61.5%)	76 (66.4%)	67 (77.0%)	16 (61.5%)	83 (73.5%)	
No information/not applicable	3 (3.5%)	0	3 (2.7%)	7 (8.1%)	1 (3.8%)	8 (7.1%)	
Wound dehiscence	not applicable			0	1 (3.8%)	1 (0.9%)	
Cancer recurrence				4 (4.6%)	5 (19.2%)	9 (8.0%)	
Chest wall inflammation				0	1 (3.8%)	1 (0.9%)	
Enlarged lymph nodes				0	1 (3.8%)	1 (0.9%)	
Pain				0	1 (3.8%)	1 (0.9%)	
Infection				2 (2.3%)	0	1 (0.9%)	
Removal of the prosthesis				4 (4.6%)	0	4 (3.5%)	
Sensory disturbances				1 (1.1%)	0	1 (0.9%)	
Decompression of fluid				1 (1.1%)	0	1 (0.9%)	
Suspected cancer metastases				3 (3.4%)	0	3 (2.7%)	
Assessment of efficacy and safety							
Effective	80 (92.0%)	22 (84.6%)	102 (90.3%)	73 (84.0%)	18 (69.2%)	91 (80.5%)	
Unsatisfactory	2 (2.3%)	2 (7.7%)	4 (3.5%)	3 (3.4%)	4 (15.4%)	7 (6.2%)	0.155 *
No information/not applicable	5 (5.7%)	2 (7.7%)	7 (6.2%)	11 (12.6%)	4 (15.4%)	15 (13.3%)	<0.001 **
Adverse events							
Abscess	0	1 (3.8%)	1 (0.9%)	not applicable			
Implant displacement	0	1 (3.8%)	1 (0.9%)				0.763
Excision of a prosthesis fragment	1 (1.1%)	0	1 (0.9%)				
Prosthesis collapse	1 (1.1%)	0	1 (0.9%)				
Infection	1 (1.1%)	0	1 (0.9%)				
Removal of the implant	2 (2.3%)	0	2 (1.8%)				
None	78 (89.7%)	22 (84.6%)	100 (88.5%)	77 (88.5%)	18 (69.2%)	95 (84.1%)	
No information available	4 (4.6%)	2 (7.7%)	6 (5.3%)	10 (11.5%)	5 (19.2%)	15 (13.3%)	
Leakage from an open wound	not applicable			0	1 (3.8%)	1 (0.9%)	0.003
Pain at the graft site				0	1 (3.8%)	1 (0.9%)	
Fistula				0	1 (3.8%)	1 (0.9%)	
Chest wall inflammation				0	1 (3.8%)	1 (0.9%)	
Assessment of pain sensation							
0	2 (2.3%)	0	2 (1.8%)	0	0	0	
1	20 (23.0%)	7 (26.9%)	27 (23.9%)	24 (27.6%)	18 (69.3%)	42 (37.2%)	
2	42 (48.4%)	16 (61.5%)	58 (51.3%)	40 (46.1%)	3 (11.5%)	43 (38.0%)	
3	3 (3.4%)	2 (7.7%)	5 (4.4%)	1 (1.1%)	2 (7.7%)	3 (2.6%)	
4	11 (12.6%)	0	11 (9.7%)	8 (9.2%)	1 (3.8%)	9 (8.0%)	<0.001
5	6 (6.9%)	0	6 (5.3%)	1 (1.1%)	0	1 (0.9%)	
7	0	0	0	2 (2.3%)	0	2 (1.8%)	
No information/not applicable	3 (3.4%)	2 (7.7%)	5 (4.4%)	11 (12.6%)	2 (7.7%)	13 (11.5%)	
Assessment of function/dysfunction							
Correct	79 (90.8%)	24 (92.4%)	103 (91.1%)	73 (84.0%)	18 (69.3%)	91 (80.5%)	
Incorrect	2 (2.3%)	1 (3.8%)	3 (2.7%)	3 (3.4%)	3 (11.5%)	6 (5.3%)	<0.001
No information/not applicable	6 (6.9%)	1 (3.8%)	7 (6.2%)	11 (12.6%)	5 (19.2%)	16 (14.2%)	
Foreign body sensation							
Yes	19 (21.8%)	1 (3.8%)	20 (17.7%)	18 (20.7%)	3 (11.5%)	21 (18.6%)	
No	64 (73.6%)	25 (96.2%)	89 (78.8%)	57 (65.5%)	22 (84.7%)	79 (69.9%)	0.046 *
No information available	4 (4.6%)	0	4 (3.5%)	12 (13.8%)	1 (3.8%)	13 (11.5%)	0.295 **

* Applies to the first follow-up visit. ** Applies to the second follow-up visit.

**Table 5:** Final interview data.

	Codubix^®^ ŻEBRA Prosthesis Implanted Using the ‘Hammock’ Method (*n* = 20)	Codubix^®^ ŻEBRA Prosthesis Implanted Using the ‘Rigid’ Method (*n* = 25)	All Patients (*n* = 45)	*p*-Value
Number of days post-surgery	991 (81–3109)	2070 (104–5165)	1568 (81–5165)	<0.001
Assessment of pain sensation				
1	8 (40%)	12 (48%)	20 (44.4%)	
2	4 (20%)	0	4 (8.9%)	
3	4 (20%)	1 (4%)	5 (11.1%)	
4	3 (15%)	0	3 (6.7%)	<0.001
6	1 (5%)	0	1 (2.2%)	
No information available	0	12 (48%)	(26.7%)	

**Table 6:** Quality of life after surgery.

	Codubix^®^ ŻEBRA Prosthesis Implanted Using the ‘Hammock’ Method (*n* = 20)	Codubix^®^ ŻEBRA Prosthesis Implanted Using the ‘Rigid’ Method (*n* = 25)	All Patients (*n* = 45)	*p*-Value
Comfort/ discomfort rating		Post-operative data		
No objections	80 (92.0%)	26 (100.0%)	106 (93.8%)	
Discomfort	5 (5.7%)	0	5 (4.4%)	0.526
No information/not applicable	2 (2.3%)	0	2 (1.8%)	
Comfort/ discomfort rating		First follow-up visit		
No objections	78 (89.7%)	24 (92.4%)	102 (90.3%)	
Discomfort	5 (5.7%)	1 (3.8%)	6 (5.3%)	0.196
No information/not applicable	4 (4.6%)	1 (3.8%)	5 (4.4%)	
Comfort/ discomfort rating		Second follow-up visit		
No objections	73 (84.0%)	18 (69.3%)	91 (80.5%)	
Discomfort	3 (3.4%)	3 (11.5%)	6 (5.3%)	0.001
No information/not applicable	11 (12.6%)	5 (19.2%)	16 (14.2%)	
Comfort/ discomfort rating		Final visit		
No objections	20 (100%)	8 (32%)	28 (62.3%)	
Discomfort	0	6 (26%)	6 (13.3%)	<0.001
No information/not applicable	0	11 (44%)	11 (24.4%)	

## Data Availability

The data presented in this study are available upon request from the corresponding author.

## Supplementary Materials

The following supporting information can be downloaded at: https://www.mdpi.com/article/10.3390/jcm15135297/s1, Table S1: Physical and mechanical properties of the Codubix^®^ S ŻEBRA prostheses; Table S2: Chemical properties of the Codubix^®^ ŻEBRA and Codubix^®^ S ŻEBRA prostheses; Table S3: Non-parametric Mann-Whitney U test of the distribution of surgery duration by type of method used (hammock/rigid); Table S4: Non-parametric Mann-Whitney U test of the distribution of surgery duration by type of method used (hammock/rigid); Table S5: Non-parametric Mann-Whitney U test of the distribution of recovery period by type of method used (hammock/rigid); Table S6: Chi-square test with Yates correction (bivariate tables) showing the incidence of complications on the first day after surgery in relation to the method used (hammock/rigid); Table S7: A Chi-square test (bivariate tables) showing the incidence of complications up to 6 months after surgery in relation to the method used (hammock/rigid); Table S8: A Chi-square test (bivariate tables) showing the incidence of complications after 6 months after surgery in relation to the method used (hammock/rigid); Table S9: Chi-square test (bivariate tables) showing the incidence of complications after 6 months after surgery in relation to the method used (hammock/rigid); Table S10: Chi-square test (bivariate tables) showing the incidence of adverse events after 6 months after surgery in relation to the method used (hammock/rigid); Figure S1: Diagram showing the evaluation of efficacy and safety up to 6 and after 6 months depending on the method of prosthesis implantation used (hammock/rigid); Figure S2: Diagram showing comfort ratings up to 6 and after 6 months, depending on the prosthesis implantation method (hammock/rigid); Figure S3: Diagram showing foreign body sensation in patients up to 6 and after 6 months depending on the prosthesis implantation method (hammock/rigid); Table S11: Confidence interval for all patients (hammock and rigid fixation method); Table S12: Confidence interval for hammock surgery patients; Table S13: Confidence interval for patients operated using the rigid method.
