# Supplementary material for: Evaluation of the Effectiveness and Safety of the Use of CODUBIX® ŻEBRA, CODUBIX® S ŻEBRA Rib Bone Prostheses
Source: J Clin Med. 2026 Jul 7;15(13):5297. doi: 10.3390/jcm15135297 (PMC13363458; doi:10.3390/jcm15135297)
Supplement: Supplementary file 1 [file jcm-15-05297-s001.zip › jcm-4305371-supplementary.pdf]

Title

Evaluation of the effectiveness and safety of the use of CODUBIX® ŻEBRA, CODUBIX® S ŻEBRA rib bone prostheses

Tadeusz Orłowski[1], Marcin Zieliński[2], Janusz Włodarczyk[3,4], Piotr Kasprzak[5], Magdalena Tokarska[6], Kaja Jezierska[7], Witold Sujka[7*]

[1] Institute of Tuberculosis and Lung Diseases, 01-138, Warsaw, Poland
[2] The Respiratory Hospital in Zakopane, 34-500, Zakopane, Poland
[3] Department of Thoracic and Surgical Oncology, John Paul II Hospital Cracow, Poland
[4] Jagiellonian University Collegium Medicum, Department of Thoracic Surgery
[5] Department of Neurosurgery, Medical University of Lodz, Lodz, Poland
[6] Lodz University of Technology, Lodz, Poland
[7] Tricomed S.A., Świętojańska 5/9, 93-493 Lodz, Poland
*Corresponding author: witold.sujka@tzmo-global.com

Corresponding author. Witold Sujka, Tricomed S.A., Świętojańska 5/9, 93-493 Łódź, Poland. **E-mail** witold.sujka@tzmo-global.com, **ORCID ID**: https://orcid.org/0000-0003-0293-6681

Supplementary Materials - Index

Supplementary Figures and Tables

Supplementary Table S1 *page 2*
Supplementary Table S2 *page 2*
Supplementary Table S3 *page 2*
Supplementary Table S4 *page 3*
Supplementary Table S5 *page 3*
Supplementary Table S6 *page 3*
Supplementary Table S7 *page 4*
Supplementary Table S8 *page 4*
Supplementary Table S9 *page 5*
Supplementary Table S10 *page 5*
Supplementary Figure S1 *page 6*
Supplementary Figure S2 *page 6*
Supplementary Figure S3 *page 7*
Supplementary Table S11 *page 7*
Supplementary Table S12 *page 7*
Supplementary Table S13 *page 8*

Supplementary Figures and Tables

Supplementary Table S1. Physical and mechanical properties of the Codubix® S ŻEBRA prostheses

No.	Tested parameter	Unit	Values											
			Feature											
			RZ-1	RZ-2	RZ-3	RZ-4	RZ-5	RZ-6	RZ-7	RZ-8	RZ-9	RZ-10	RZ-11	RZ-12
1.	dimensions (a x b or a x b x c)	mm	250 x 20	250 x 200	250 x 65	200 x 125	31 x 63 x 53	30 x 76 x 70	42 x 91 x 81	24 x 44 x 93	30 x 60 x 117	34 x 71 x 130	42 x 79 x 133	37 x 89 x 161
2.	thickness	mm	5.000						3.300					

Supplementary Table S2. Chemical properties of the Codubix® ŻEBRA and Codubix® S ŻEBRA prostheses

No.	Parameter	Unit	Value
1.	organoleptic evaluation of the extract<br>transparency<br>colour	-<br>-	transparent<br>colourless
2.	pH of the tested sample	pH unit	5.5–8.0
3.	permanganate oxidisability	mg O_2/g	max. 0.08
4.	max. absorbance in the ultraviolet wavelength range: 220–360 nm	Amax (nm)	max. 0.3
5.	foaming agents	foam height (cm)	absent
6.	content of substances soluble in petroleum ether	%	max. 1.5

Supplementary Table S3. Non-parametric Mann-Whitney U test of the distribution of surgery duration by type of method used (hammock/rigid)

**Variable**	**Rank sum Hammock method**	**Rank sum Rigid method**	**U**	**Z**	**p-value**	**Z adjusted**	**p-value***
Duration of surgery	5122.0	1319.0	968.000	1.109	0.268	1.114	0.265

*p-value used in statistical hypothesis testing to determine if a result is statistically significant

Supplementary Table S4. Non-parametric Mann-Whitney U test of the distribution of surgery duration by type of method used (hammock/rigid)

Variable	Rank sum Hammock method	Rank sum Rigid method	U	Z	p-value	Z adjusted	p-value*
Hospitalisation time	5496.5	944.5	593.500	3.663	0.0002	3.667	0.0002

*p-value used in statistical hypothesis testing to determine if a result is statistically significant

Supplementary Table S5. Non-parametric Mann-Whitney U test of the distribution of recovery period by type of method used (hammock/rigid)

Variable	Rank sum Hammock method	Rank sum Rigid method	U	Z	p-value	Z adjusted	p-value*
Recovery period	1903.5	4537.5	709.500	-2.872	0.004	-2.985	0.003

*p-value used in statistical hypothesis testing to determine if a result is statistically significant

Supplementary Table S6. Chi-square test with Yates correction (bivariate tables) showing the incidence of complications on the first day after surgery in relation to the method used (hammock/rigid)

Statistic: Chi-2 Yates	df	p-value	
1.795	df=1	p=0.180	
Complications in the first days after the surgery	Hammock method	Rigid method	Row totals
No	68	24	92
Column %	78.16%	92.31%	
Row %	73.91%	26.09%	
Total %	60.18%	21.24%	81.42%
Yes	19	2	21
Column %	21.84%	7.69%	
Row %	90.48%	9.52%	
Total %	16.81%	1.77%	18.58%
Totals	87	26	113
Total %	76.99%	23.01%	100.00%

Supplementary Table S7. A Chi-square test (bivariate tables) showing the incidence of complications up to 6 months after surgery in relation to the method used (hammock/rigid).

Statistic: Chi-2 Pearson	df	p-value	
1.848	df=2	p=0.397	
Complications up to 6 months	Hammock method	Rigid method	Row totals
None	60	16	76
Column %	68.97%	61.54%	
Row %	78.95%	21.05%	
Total %	53.10%	14.16%	88.50%
Yes	24	10	34
Column %	27.59%	38.46%	
Row %	70.59%	29.41%	
Total %	21.24%	8.85%	30.09%
No information/not applicable	3	0	3
Column %	3.45%	0.00%	
Row %	100.00%	0.00%	
Total %	2.65%	0.00%	2.65%
Totals	87	26	113
Total %	76.99%	23.01%	100.00%

Supplementary Table S8. A Chi-square test (bivariate tables) showing the incidence of complications after 6 months after surgery in relation to the method used (hammock/rigid).

Statistic: Chi-2 Pearson	df	p-value	
5.130	df=2	p=0.077	
Complications after 6 months	Hammock method	Rigid method	Row totals
None	67	16	83
Column %	77.01%	61.54%	
Row %	80.72%	19.28%	
Total %	59.29%	14.16%	73.45%
Yes	13	9	22
Column %	14.94%	34.62%	
Row %	59.09%	40.91%	
Total %	11.50%	7.96%	19.47%
No information/not applicable	7	1	8
Column %	8.05%	3.85%	
Row %	87.50%	12.50%	
Total %	6.19%	0.88%	7.08%
Totals	87	26	113
Total %	76.99%	23.01%	100.00%

Supplementary Table S9. Chi-square test (bivariate tables) showing the incidence of complications after 6 months after surgery in relation to the method used (hammock/rigid).

Statistic: Chi-2 Pearson	df	p-value	
0.541	df=2	p=0.763	
Adverse events up to 6 months	Hammock method	Rigid method	Row totals
None	78	22	100
Column %	89.66%	84.62%	
Row %	78.00%	22.00%	
Total %	69.03%	19.47%	88.50%
Yes	5	2	7
Column %	5.75%	7.69%	
Row %	71.43%	28.57%	
Total %	4.42%	1.77%	6.19%
No information/not applicable	4	2	6
Column %	4.60%	7.69%	
Row %	66.67%	33.33%	
Total %	3.54%	1.77%	5.31%
Totals	87	26	113
Total %	76.99%	23.01%	100.00%

Supplementary Table S10. Chi-square test (bivariate tables) showing the incidence of adverse events after 6 months after surgery in relation to the method used (hammock/rigid).

Statistic: Chi-2 Pearson	df	p-value	
11.826	df=2	p=0.003	
Adverse events after 6 months	Hammock method	Rigid method	Row totals
None	77	18	95
Column %	88.51%	69.23%	
Row %	81.05%	18.95%	
Total %	68.14%	15.93%	84.07%
Yes	0	3	3
Column %	0.00%	11.54%	
Row %	0.00%	100.00%	
Total %	0.00%	2.65%	2.65%
No information/not applicable	10	5	15
Column %	11.49%	19.23%	
Row %	66.67%	33.33%	
Total %	8.85%	4.42%	13.27%
Totals	87	26	113
Total %	76.99%	23.01%	100.00%

Supplementary Figure S1. Diagram showing the evaluation of efficacy and safety up to 6 and after 6 months depending on the method of prosthesis implantation used (hammock/rigid)

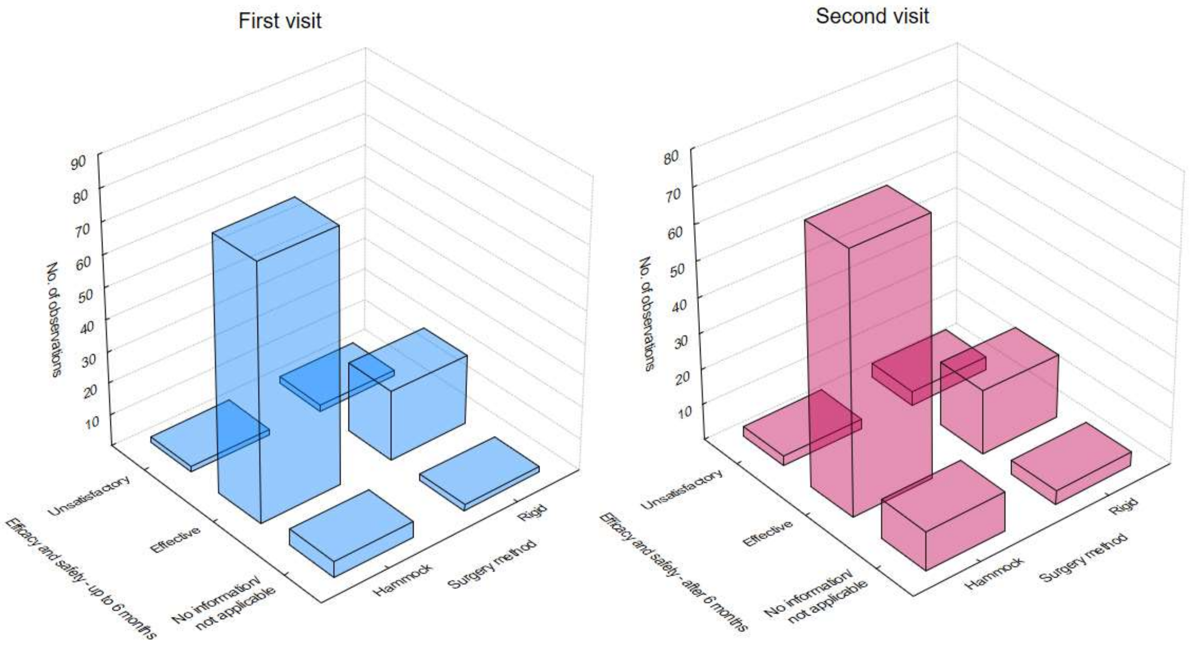

Supplementary Figure S2. Diagram showing comfort ratings up to 6 and after 6 months, depending on the prosthesis implantation method (hammock/rigid)

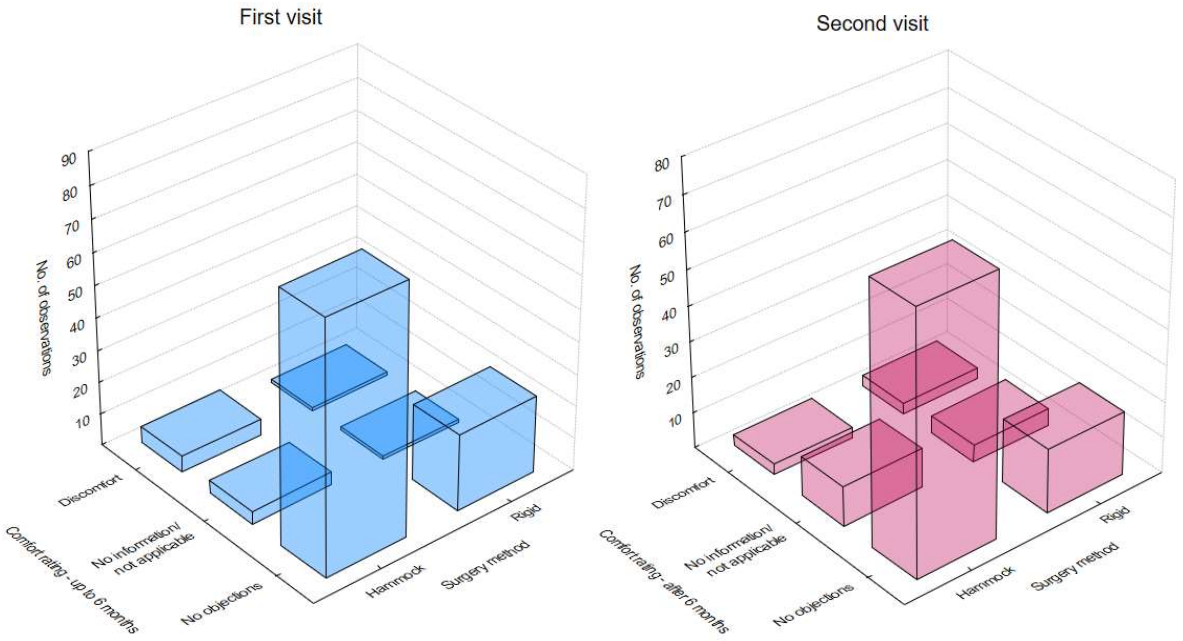

Supplementary Figure S3. Diagram showing foreign body sensation in patients up to 6 and after 6 months depending on the prosthesis implantation method (hammock/rigid)

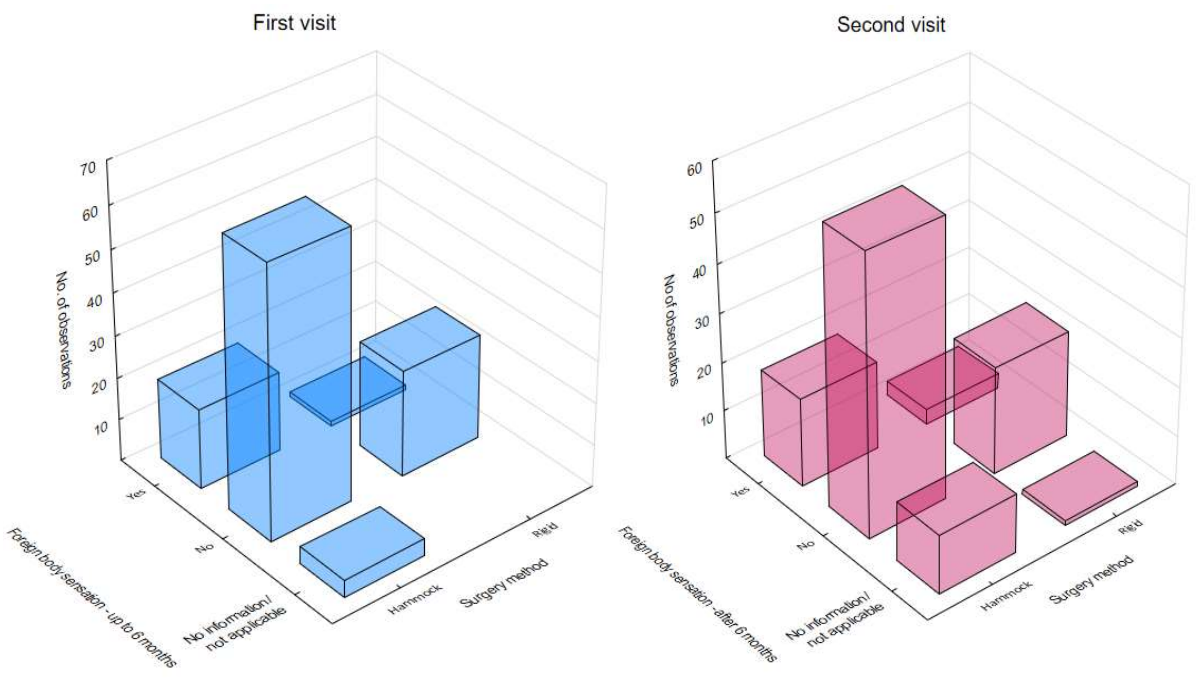

Supplementary Table S11. Confidence interval for all patients (hammock and rigid fixation method)

	Hammock + Rigid					
	N-valid	**Mean**	**Confidence −95.000%**	**Confidence +95.000%**	median	standard deviation
Age on the day of surgery, years	113	53	50	56	56	16
Duration of surgery, min.	112	218	206	231	228	66
Hospitalisation time, days	113	21	18	24	15	16
Recovery, weeks	95	8	7	8	8	4

Supplementary Table S12. Confidence interval for hammock surgery patients

	Hammock					
	N-valid	**Mean**	**Confidence −95.000%**	**Confidence +95.000%**	median	standard deviation
Age on the day of surgery, years	87	52	49	56	56	15
Duration of surgery, min.	87	217	202	232	225	70
Hospitalisation time, days	87	23	20	27	18	17
Recovery, weeks	84	8	7	9	8	4

Supplementary Table S13. Confidence interval for patients operated using the rigid method

	Rigid					
	N-valid	**Mean**	**Confidence −95.000%**	**Confidence +95.000%**	median	standard deviation
Age on the day of surgery, years	26	53	46	61	62	18
Duration of surgery, min.	25	223	203	244	240	50
Hospitalisation time, days	26	14	10	19	11	11
Recovery, weeks	11	4	4	5	4	1
